# Supplementary material for: Policies and strategies for HPV vaccination schedule completion in immunocompromised girls, including girls living with HIV: Qualitative insights from Eswatini, Malawi, and Uganda
Source: PLOS Glob Public Health. 2026 Feb 3;6(2):e0004688. doi: 10.1371/journal.pgph.0004688 (PMC12867219; doi:10.1371/journal.pgph.0004688)
Supplement: S1 Text — (DOCX) [file pgph.0004688.s001.docx]

Interview Guides

Contents

[Eswatini 2](#_Toc206754982)

[Key Informant Interview Guide 2](#_Toc206754983)

[Focus Group Discussion Guide 5](#_Toc206754984)

[Malawi 8](#_Toc206754985)

[Key Informant Interview Guide 8](#_Toc206754986)

[Focus group discussion Guide 11](#_Toc206754987)

[Uganda 19](#_Toc206754988)

[Key Informant Interview Guide 19](#_Toc206754989)

[Focus group discussion Guide 22](#_Toc206754990)

# Eswatini

## Key Informant Interview Guide

Date: ___/___/___

**Stakeholder group:** National and Subnational level stakeholders

**Interviewer:** Thank you for agreeing to participate in this interview. As a reminder, this interview

should take approximately 30-45 minutes to complete, and there are no right or wrong answers. We

would like your insights on the implementation of HPV vaccination policies for People Living with HIV

(PLHIV) especially HIV+ and immunocompromised girls (also known as adolescent girls with HIV). These interviews will be conducted with stakeholders, like yourself who play a key role at the national or subnational level on HPV vaccination program.

If you agree, your interview will be audio recorded and stored on a secure server. No individual data

will be released or made public in any way. Your responses will remain confidential, and your name

will not be associated with your responses. You do not have to answer every question, and you can

end the interview at any time. Your participation in this interview is completely voluntary. Is it ok

if we audio record?

Do you have any questions before we start? [Answer questions.]

The interviewer administers consent form and interviewee signs before the interview starts.

**Key Informant Interview:**

Organization:­­­­­­­­­_________________

Designation of Stakeholders: _____________

Age: _____________

Gender: ­­­­­­______________

Number of years in healthcare services:­­­­____________

Date:­­­___/___/____

Identifier: ___________

**A. General Knowledge on HPV vaccination**

1. When was HPV vaccine introduced in your country? Month and year.
2. What is the HPV Vaccine? Do you know the disease it protects against?
3. What was your role during the HPV vaccine introduction?
4. How many doses of HPV vaccines are given to adolescent girls?
5. How many doses are given to adolescent girls living with HIV?
6. Since HPV vaccine introduction, what strategies have been implemented to vaccinate adolescent girls? Probe on school based, facility based and community-based strategies.

**B. Policies, strategies and practices for vaccinating HIV positive individuals**

1. Specifically for adolescent girls with HIV, what are the strategies implemented to vaccinate this cohort?
   - 1. Probe on strategies for identifying and vaccinating HIV+ and immunocompromised girls. Are HPV vaccines delivered in ART clinics? If yes, probe on who is responsible and how vaccines are received and stored.
     2. Probe for the approach to follow up on adolescent girls with HIV to get them fully vaccinated.
   1. In your opinion, are there any specific programs or initiatives aimed at improving HPV vaccination rates among HIV+ and immunocompromised girls (adolescent girls with HIV)? If so, can you describe them?
2. In your opinion, what are or could be effective ways of reaching HIV positive individuals

for complete HPV vaccination in your country?

**C.  Assessing perception (awareness, feasibility, and acceptability) of vaccinating HIV positive individuals**

1. In your words, briefly explain why HPV vaccination is especially important for adolescents living with HIV.
2. From your perspective, how would you rate the sufficiency of the current strategies towards reaching HIV+ and immunocompromised girls with HPV vaccines. Please rate from 1-5 to rank sufficiency with 1- Not sufficient at all, 2 - Not sufficient, 3 -Neutral 4- Somewhat sufficient, 5 - Very/completely sufficient.
3. In your opinion, briefly explain the factors influencing HPV vaccination in HIV+ and immunocompromised girls (adolescent girls with HIV).
4. Have you noticed any changes in the attitudes and perceptions of different stakeholders towards the HPV vaccination for HIV+ and immunocompromised girls (adolescent girls with HIV)?
5. Are there other initiatives beyond those mentioned above that influenced the acceptability and uptake of the HPV vaccines in HIV+ and immunocompromised girls? Kindly elaborate

**D.** **Barriers and facilitators to HPV vaccination** **in HIV positive individuals**

1. From your perspective, what are the main challenges Ministry of Health providers face in vaccinating HIV+ and immunocompromised girls against HPV?
2. Probe: How do logistical issues, such as vaccine availability and access to healthcare facilities or schools, affect HPV vaccination rates among HIV positive girls and immunocompromised girls (adolescent girls with HIV)?
3. In your opinion, can you describe any misconceptions or fears about the HPV vaccine amongst parents or guardians of HIV positive girls?
4. How do logistical issues, such as vaccine availability and access to healthcare facilities, affect HPV vaccination rates among HIV positive girls?
5. Are there any specific factors that have made vaccination of HIV positive individuals in your setting successful?
6. Probe: if yes, please explain these factors
7. Are there any programs that enabled the vaccination of HIV positive individuals?
8. In your opinion, what are some of the best practices or successful strategies that have been reported so far from the field in the implementation of HPV vaccination in HIV+ individuals?
9. Probe: Can you share some specific examples of successful interventions or initiatives that have positively influenced the acceptability and uptake of the HPV vaccine in HIV positive individuals?

**E. Assessing opportunities for better integration of HPV vaccination for HIV positive individuals at scale.**

1. Based on your insights and experience, what other recommendations would you make to sustain and further increase HPV vaccination coverage among HIV positive individuals in your country? Probe on strategies for reaching through facility, school and community strategies.
2. Are there any additional insights or recommendations you would like to share regarding HPV vaccination in HIV positive individuals?

## Focus Group Discussion Guide

Date: ­­­­___/___/____

| **Designation of HCW** | **Gender** | **Name of Facility** | **Setting (Urban/Rural)** |
| --- | --- | --- | --- |
|  |  |  |  |
|  |  |  |  |
|  |  |  |  |
|  |  |  |  |
|  |  |  |  |
|  |  |  |  |
|  |  |  |  |
|  |  |  |  |
|  |  |  |  |

**A. General Knowledge of HPV vaccination**

1. Can anyone share what they know about HPV vaccination? And the diseases hat HPV vaccine can protect?
2. When was HPV vaccine introduced in your facility? Month and year
3. How many doses of HPV vaccines are given to adolescent girls?
4. If a single dose, probe to know how many doses are given to HIV positive individuals.
5. How many doses of HPV vaccine are given to HIV+ and immunocompromised girls (adolescent girls with HIV)? If country has adopted a single dose schedule, probe if the guidelines for doses to adolescent girls living with HIV has changed with the adoption of single dose HPV schedule, perhaps from 3 dose to 2 dose.
6. Since HPV vaccine introduction, what strategies have been implemented to vaccinate adolescent girls? Probe on school based, facility based and community-based strategies.

**B. Policies, strategies and practices for vaccinating HIV positive individuals**

1. Can anyone share the strategies for vaccinating HIV positive individuals or immunocompromised individuals (adolescent girls wit HIV)?
   1. Probe on strategies for identifying and vaccinating HIV+ and immunocompromised girls. Are HPV vaccines delivered in ART clinics? If yes, probe on who is responsible and how vaccines are received and stored
   2. If country introduced a single dose, probe for the approach to follow up on HIV+ and immunocompromised girls (adolescent girls with HIV) and get them fully vaccinated.
   3. If the country is yet to adopt single dose schedule, probe for the approach to completely vaccinate HIV+ and immunocompromised girls (adolescent girls with HIV) if different.
2. Are there any specific programs or initiatives aimed at improving HPV vaccination rates among HIV + and immunocompromised girls? If so, can you describe them?
3. Are there any promising practices for HPV vaccination for adolescent girls with HIV that you are aware of in your country? If so, can you describe them?

**C.  Assessing perception (awareness, feasibility, and acceptability) of vaccinating HIV positive individuals**

1. In your words, briefly explain why HPV vaccination is especially important for adolescents living with HIV?
2. In your opinion, briefly explain the factors influencing HPV vaccination in HIV+ and immunocompromised girls (adolescent girls with HIV)?
3. Have you noticed any changes in the attitudes and perceptions of different stakeholders towards the HPV vaccination for HIV positive individuals?
4. Are there other initiatives beyond those mentioned above that influenced the acceptability and uptake of the HPV vaccines? Kindly elaborate.

**D.** **Barriers and facilitators to HPV vaccination** **in HIV positive individuals**

1. From your perspective, what are the main challenges for Ministry of Health (Programs and healthcare workers) in vaccinating HIV positive girls against HPV?
2. Probe: How do logistical issues, such as vaccine availability and access to healthcare facilities, affect HPV vaccination rates among HIV positive girls?
3. In your opinion, can you describe any misconceptions or fears about the HPV vaccine amongst parents or guardians of Adolescents Living with HIV (ALHIV)?
4. In your opinion, are there any barriers relating to gender in vaccinating ALHIV?
5. How do logistical issues, such as vaccine availability and access to healthcare facilities, affect HPV vaccination rates among HIV positive girls?
6. How do HIV related issues, such as issues related to navigating the complexities of managing both disclosure and HIV treatment, stigma, including internalized stigma, and discrimination, affect HPV vaccination rates among ALHIV girls?
7. Are there any specific factors that have encouraged parents/caregivers or adolescents to bring their child or self to the facility for vaccination?
   - 1. Probe: if yes, please explain these factors
     2. Are there any programs that enabled the vaccination of HIV+ and immunocompromised girls (adolescent girls with HIV)?
     3. Are there any communication or social mobilization strategies that promoted awareness about HPV vaccination in HIV+ and immunocompromised girls (adolescent girls with HIV)?

7. In your opinion, what are some of the best practices or successful strategies that have been reported so far from the field in the implementation of HPV vaccination in HIV+ and immunocompromised girls (adolescent girls with HIV)?

- - 1. Probe: Can you share some specific examples of successful interventions or initiatives that have positively influenced the acceptability and uptake of the HPV vaccine in HIV+ and immunocompromised girls (adolescent girls with HIV)?

**E. Assessing opportunities for better integration of HPV vaccination for HIV positive individuals at scale.**

1. Based on your insights and experience, what recommendations would you make to sustain and further increase HPV vaccination coverage among HIV positive individuals in your country?
2. Is there any other thing anyone wants to share regarding HPV vaccination in HIV positive individuals?

# Malawi

## Key Informant Interview Guide

**Stakeholder group:** National and Subnational level stakeholders

**Interviewer:** Thank you for agreeing to participate in this interview. As a reminder, this interview should take approximately 30-45 minutes to complete, and there are no right or wrong answers. We would like your insights on the implementation of HPV vaccination policies for People Living with HIV (PLHIV), especially HIV+ and immunocompromised girls (also known as adolescent girls with HIV). These interviews will be conducted with stakeholders, like yourself who play a key role at the national or subnational level in the HPV vaccination program.

If you agree, your interview will be audio-recorded and stored on a secure server. No individual data will be released or made public in any way. Your responses will remain confidential, and your name will not be associated with your responses. You do not have to answer every question, and you can end the interview at any time. Your participation in this interview is completely voluntary. Is it ok if we audio record?

Do you have any questions before we start? [Answer questions.]

The interviewer administers the consent form and the interviewee signs before the interview starts.

KII Interview questions:

**Organization: _____________________________**

**Designation of Stakeholder: ____________________**

**Age: ___________________**

**Gender: _________________**

**Number of years in healthcare service:**

**Date: ____ /____ /____**

**Identifier code:**

**A. General Knowledge on HPV vaccination**

1. What is HPV vaccine? Do you know the diseases it protects against?
2. When was HPV vaccine introduced in your country? Month and year
3. What was your role during the HPV vaccine introduction?
4. How many doses of HPV vaccines are given to adolescent girls?
5. How many doses are given to adolescents girls living with HIV? If country has adopted a single dose schedule, probe if the guidelines for doses to adolescent girls living with HIV has changed with the adoption of single dose HPV schedule, perhaps from 3 dose to 2 dose? (PRISM: Evaluating Intervention Characteristics)
6. Since HPV vaccine introduction, what strategies have been implemented to vaccinate adolescent girls? Probe on school based, facility based and community based strategies. (PRISM: Evaluating Intervention Characteristics)

**B. Policies, strategies, and practices for vaccinating adolescent girls with HIV**

1. Specifically, for adolescent girls with HIV, what are the strategies implemented to vaccinate this cohort? (PRISM: Evaluating Intervention Characteristics)
   1. Probe on strategies for specifically identifying and vaccinating HIV+ and immunocompromised girls vs general population. Are HPV vaccines delivered in ART clinics? If yes, probe on who is responsible and how vaccines are received and stored.
   2. If the country introduced a single dose, probe for the approach to follow up on adolescent girls with HIV to get them fully vaccinated.
   3. If the country is yet to adopt single dose schedule, probe for the approach to completely vaccinate HIV+ and immunocompromised girls (adolescent girls with HIV) if different.
2. To your knowledge, are there any specific programs or initiatives aimed at improving HPV vaccination rates among HIV+ and immunocompromised girls (adolescent girls with HIV)? If so, can you describe them? (PRISM: Assessing External Environment)
3. In your opinion, are there any promising practices for HPV vaccination for adolescent girls with HIV that you are aware of in your country? (PRISM: Assessing External Environment)

C**. Assessing perception (awareness, feasibility, and acceptability) of vaccinating HIV+ and immunocompromised girls (adolescent girls with HIV)**

1. In your words, briefly explain why HPV vaccination is especially important for adolescents living with HIV

From your perspective, how would you rate the sufficiency of the current strategies towards reaching HIV+ and immunocompromised girls with HPV vaccines. Please rate from 1-5 to rank sufficiency with 1- Not sufficient at all, 2 - Not sufficient, 3 -Neutral 4- Somewhat sufficient, 5 - Very/completely sufficient (PRISM: Assessing External Environment)

In your opinion, briefly explain the factors influencing HPV vaccination in HIV+ and immunocompromised girls (adolescent girls with HIV). (PRISM: Assessing External Environment)

1. How do different stakeholders perceive HPV vaccination in ALHIV?
   1. Probe: Are there differences among women vs men, healthcare workers vs school admin/teachers vs parents vs others?

3. Are there other initiatives beyond those mentioned above that influenced the acceptability and uptake of the HPV vaccines in HIV+ and immunocompromised girls? Kindly elaborate (PRISM: Assessing External Environment)

1. From your perspective, how would you rate the feasibility of implementing effective strategies for reaching HIV+ and immunocompromised girls with HPV vaccines in your country. Please rate from 1-5 to rank feasibility with 1- Not feasible at all, 2 - Not feasible, 3 -Neutral 4- Somewhat feasible, 5 - Very/completely feasible (PRISM: Evaluating Intervention Characteristics)

D. **Barriers and facilitators to HPV vaccination** **in HIV+ and immunocompromised girls (adolescent girls with HIV)**

1. From your perspective, what are the main challenges EPI/healthcare providers face in vaccinating HIV+ and immunocompromised girls (adolescent girls with HIV) against HPV?
   1. Probe: How do logistical issues, such as vaccine availability and access to healthcare facilities, affect HPV vaccination rates among HIV+ and immunocompromised girls (adolescent girls with HIV)? (PRISM: Assessing External Environment)
2. In your opinion, can you describe any misconceptions or fears about the HPV vaccine amongst parents or guardians of HIV+ and immunocompromised girls (adolescent girls with HIV)? (PRISM: Understanding Recipient Characteristics)
3. In your opinion, are there social or cultural norms that influence ALHIV vaccination? (PRISM: Assessing External Environment)
4. How do logistical issues, such as vaccine availability and access to healthcare facilities, affect HPV vaccination rates among HIV+ and immunocompromised girls (adolescent girls with HIV)? (PRISM: Assessing External Environment)
5. Are there any specific factors that have facilitated the vaccination of HIV+ and immunocompromised girls (adolescent girls with HIV) with the HPV vaccine in your setting? (PRISM: Assessing External Environment)
   1. Probe: if yes, please explain these factors
   2. Are there any programs that enabled the vaccination of HIV+ and immunocompromised girls (adolescent girls with HIV)?
6. In your opinion, what are some of the best practices or successful strategies that have been reported so far from the field in the implementation of HPV vaccination in HIV+ and immunocompromised girls (adolescent girls with HIV)? (PRISM: Assessing External Environment)
   1. Probe: Can you share some specific examples of successful interventions or initiatives that have positively influenced the acceptability and uptake of the HPV vaccine in HIV+ and immunocompromised girls (adolescent girls with HIV)?

**E. Assessing opportunities for better integration of HPV vaccination for HIV+ and immunocompromised girls (adolescent girls with HIV) at scale.**

1. Based on your insights and experience, what other recommendations would you make to sustain and further increase HPV vaccination coverage among HIV+ and immunocompromised girls (adolescent girls with HIV) in your country? Probe on strategies for reaching through facility, school and community strategies (PRISM: Assessing implementation and sustainability infrastructure)

a. Are there any additional insights or recommendations you would like to share regarding HPV vaccination in HIV+ and immunocompromised girls (adolescent girls with HIV)?

## Focus group discussion Guide

**Date: ____ /____ /____**

| **Designation of HCW** | **Gender** | **Name of Facility** | **Setting (urban/rural)** | **Identifier code** |
| --- | --- | --- | --- | --- |
|  |  |  |  |  |
|  |  |  |  |  |
|  |  |  |  |  |
|  |  |  |  |  |
|  |  |  |  |  |
|  |  |  |  |  |
|  |  |  |  |  |
|  |  |  |  |  |
|  |  |  |  |  |

**A. General Knowledge on HPV vaccination**

1. Can anyone share what they know about HPV vaccination? And the diseases that HPV vaccine can protect against? How it might be different for girls with HIV? (PRISM: Evaluating Intervention Characteristics)
2. When was HPV vaccine introduced in your facility? Month and year (PRISM: Evaluating Intervention Characteristics)
3. How many doses of HPV vaccines are given to adolescent girls? (PRISM: Evaluating Intervention Characteristics)
4. How many doses of HPV vaccines are given to HIV+ and immunocompromised girls (adolescent girls with HIV)? If country has adopted a single dose schedule, probe if the guidelines for doses to adolescent girls living with HIV has changed with the adoption of single dose HPV schedule, perhaps from 3 dose to 2 dose? (PRISM: Evaluating Intervention Characteristics)
5. Since HPV vaccine introduction, what strategies have been implemented to vaccinate adolescent girls? Probe on school based, facility based and community based strategies (PRISM: Evaluating Intervention Characteristics)

**B. Policies, strategies and practices for vaccinating HIV+ and immunocompromised girls (adolescent girls with HIV)**

1. Can anyone share the strategies for vaccinating HIV+ and immunocompromised girls (adolescent girls with HIV)? (PRISM: Evaluating Intervention Characteristics)
   1. Probe on strategies for specifically identifying and vaccinating HIV+ and immunocompromised girls vs general population. Are HPV vaccines delivered in ART clinics? If yes, probe on who is responsible and how vaccines are received and stored (PRISM: Evaluating Intervention Characteristics)
   2. If country introduced a single dose, probe for the approach to follow up on HIV+ and immunocompromised girls (adolescent girls with HIV) and get them fully vaccinated. (PRISM: Evaluating Intervention Characteristics)
   3. If the country is yet to adopt single dose schedule, probe for the approach to completely vaccinate HIV+ and immunocompromised girls (adolescent girls with HIV) if different. (PRISM: Evaluating Intervention Characteristics)
2. To your knowledge, are there any specific programs or initiatives aimed at improving HPV vaccination rates among HIV + and immunocompromised girls? If so, can you describe them? (PRISM: Assessing External Environment)
3. Are there any promising practices for HPV vaccination for adolescent girls with HIV that you are aware of in your country? If so, can you describe them? (PRISM: Assessing External Environment)

C**. Assessing perception (awareness, feasibility, and acceptability) of vaccinating HIV+ and immunocompromised girls (adolescent girls with HIV)**

1. In your words, briefly explain why HPV vaccination is especially important for adolescents living with HIV
2. In your opinion, briefly explain the factors influencing HPV vaccination in HIV+ and immunocompromised girls (adolescent girls with HIV)?
3. How do different stakeholders perceive HPV vaccination in ALHIV?
   1. Probe: Are there differences among women vs men, healthcare workers vs school admin/teachers vs parents vs others?
4. Are there other initiatives beyond those mentioned above that influenced the acceptability and uptake of the HPV vaccines in HIV+ and immunocompromised girls? Kindly elaborate (PRISM: Assessing External Environment)

D. **Barriers and facilitators to HPV vaccination in HIV+ and immunocompromised girls (adolescent girls with HIV)**

1. From your perspective, what are the main challenges EPI/healthcare providers face in vaccinating adolescent girls against HPV?
   1. Probe: How do logistical issues, such as vaccine availability and access to healthcare facilities, affect HPV vaccination rates among adolescent girls?
2. In your opinion, can you describe any misconceptions or fears about the HPV vaccine amongst parents or guardians of ALHIV? (PRISM: Assessing External Environment)
3. In your opinion, are there social or cultural norms that influence ALHIV vaccination? (PRISM: Assessing External Environment)
4. How do logistical issues, such as vaccine availability and access to healthcare facilities, affect HPV vaccination rates among ALHIV girls? (PRISM: Assessing External Environment)
5. How do HIV related issues, such as issues related to navigating the complexities of managing both disclosure and HIV treatment, stigma, including internalized stigma, and discrimination, affect HPV vaccination rates among ALHIV girls? (PRISM: Assessing External Environment)
6. Are there any specific factors that have encouraged parents/caregivers or adolescents to bring their child or self to the facility for vaccination? (PRISM: Assessing External Environment)
   1. Probe: if yes, please explain these factors
   2. Are there any programs that enabled the vaccination of HIV+ and immunocompromised girls (adolescent girls with HIV)?
   3. Are there any communication or social mobilization strategies that promoted awareness about HPV vaccination in HIV+ and immunocompromised girls (adolescent girls with HIV)?
7. In your opinion, what are some of the best practices or successful strategies that have been reported so far from the field in the implementation of HPV vaccination in HIV+ and immunocompromised girls (adolescent girls with HIV)? (PRISM: Assessing implementation and sustainability infrastructure)
   1. Probe: Can you share some specific examples of successful interventions or initiatives that have positively influenced the acceptability and uptake of the HPV vaccine in HIV+ and immunocompromised girls (adolescent girls with HIV)?

**E. Assessing opportunities for better integration of HPV vaccination for HIV+ and immunocompromised girls (adolescent girls with HIV) at scale.**

1. Based on your insights and experience, what recommendations would you make to sustain and further increase HPV vaccination coverage among HIV+ and immunocompromised girls (adolescent girls with HIV) in your country?
2. Is there any other thing anyone wants to share regarding HPV vaccination in HIV+ and immunocompromised girls (adolescent girls with HIV)?

**Scoping of Policies and Strategies for Reaching HIV Postive Girls for HPV Vaccine Schedule Completion: Insights from Uganda**

**Study Protocol tools: July 2024**

**GENERAL GUIDE FOR CONDUCTING KEY INFORMANT INTERVIEWS**

**Stakeholder group:** National and Subnational level stakeholders

***Introduction***

This guide is designed to facilitate key informant qualitative interviews with key stakeholders involved in the implementation of HPV vaccination policies for PLHIV in Uganda.The aim is to gather in-depth insights into the policies and practices surrounding the administration of the HPV vaccine.

**Preparing for the Interview**

- **Review Existing Data:** Before conducting interviews, gather and review any existing data on HPV vaccination policies for PLHIV particularly HIV + and immunocompromised girls .
- **Determine Information Needs**: Identify specific information gaps that the interviews should address.
- **Select Key Informants:** Choose a diverse group of stakeholders with firsthand knowledge of the policy implementation, such as healthcare providers, policymakers, and community leaders.
- **Interview Type:** Conduct the KII in person (ie face-to-face) or virtually via Zoom or Microsoft Teams. The method to adopt for KII interviews is based on logistics and informant preferences. Interviewers are encouraged to conduct face-to-face interviews for the focus group discussions.

***Conducting the Interview***

- **Introduction:** Begin by introducing yourself and explaining the purpose of the interview. Ensure the participant is informed and consents to the interview process.
- **Use of KII Guide:** Follow the interview guide to ensure all relevant topics are covered. However, be prepared to diverge from the guide to explore new lines of inquiry as they arise. It is important to get respondents to provide details and not just yes/no answers. General probes that can be used to get individuals to elaborate on the below question are:
- Can you tell me more?
- Can you give me an example?
- How do you feel about that?
- **Data Collection:** For Key informant interviews and Focus group discussions, interviews will be audio-recorded with the participant's consent. In addition, the interviewer will also take detailed notes during the interview to capture salient information to complement the recording.
- **Role of Interviewer:** Keep the interview on track, be sensitive to non-verbal cues, and provide prompts to facilitate the interviewee's responses**.**

**Key Areas of Focus**

- **Policy and Practice Implementation:** Explore how policies are translated into practice at the national and sub-national levels, including the role of policy actors in the implementation process.
- **Barriers and Facilitators:** Identify any barriers or facilitators to the implementation of HPV vaccination policies for HIV+ and immunocompromised girls**.**
- **Stakeholder Perspectives:** Gather diverse perspectives on the motivation and beliefs surrounding HPV vaccination for HIV+ and immunocompromised girls.

**Post-Interview**

- **Data Compilation:** Organize and compile the data collected from the interviews for analysis.
- **Debrief:** Consider providing a debrief to the interviewee if necessary

**Ethical Considerations**

- **Confidentiality**: Assure participants that their responses will be confidential and reported in aggregate form
- **Informed Consent:** Ensure that participants are fully informed about the study and have given their consent before proceeding with the interview.

The interview guide serves as a structured framework to ensure comprehensive coverage of the topic while allowing for the flexibility needed to capture rich, qualitative data. Interviewers should adhere to the guide but also be prepared to follow the conversation as it naturally evolves.

**KEY INFORMANT INTERVIEW GUIDE**

**Stakeholder group:** National and Subnational level stakeholders

**Interviewer:** Thank you for agreeing to participate in this interview. As a reminder, this interview should take approximately 30-45 minutes to complete, and there are no right or wrong answers. We would like your insights on the implementation of HPV vaccination policies for People Living with HIV (PLHIV) especially HIV+ and immunocompromised girls (also known as adolescent girls with HIV). These interviews will be conducted with stakeholders, like yourself who play a key role at the national or subnational level in the HPV vaccination program.

If you agree, your interview will be audio-recorded and stored on a secure server. No individual data will be released or made public in any way. Your responses will remain confidential, and your name will not be associated with your responses. You do not have to answer every question, and you can end the interview at any time. Your participation in this interview is completely voluntary. Is it ok if we audio record?

Do you have any questions before we start? [Answer questions.]

The interviewer administers the consent form and the interviewee signs before the interview starts.

KII Interview questions:

**Organization: _____________________________**

**Designation of Stakeholder: ____________________**

**Age: ___________________**

**Gender: _________________**

**Number of years in healthcare service:**

**Date: ____ /____ /____**

**Identifier code:**

1. **General Knowledge on HPV vaccination:** *The goal of this study is to learn about the policies and strategies that may help improve HPV vaccination completion for HIV + and immunicompromised gilrs in Uganda. We want to learn from your experience that you have with either general or HPV vaccine specific strategies that might work to improve HPV vaccination completion for HIV + and immunicompromised gilrs in Uganda.*
2. Could you explain your involvement or the role your department / ministry played during the introduction of the HPV vaccine in Uganda? ( Probe for the month and year HPV vaccine was introduced in Uganda)
3. How familiar are you with the specific number of doses recommended for adolescent girls in Uganda. ( Probe for current dosing schedule for HPV vaccination among adolescent girls in Uganda?)
4. In your experience, how does the dosing schedule for HPV vaccination differ for adolescent girls living with HIV? Uganda is in the process of adopting a single-dose schedule for the HPV vaccine, and I'd like to understand what considerations are being made as the country prepares for this upcoming switch. (Probe if the guidelines for doses to adolescent girls living with HIV will change with the adoption of single dose HPV schedule? (PRISM: Evaluating Intervention Characteristics).
5. Since HPV vaccine introduction, what strategies have been implemented to vaccinate adolescent girls? Probe on school based, facility based and community based strategies. (PRISM: Evaluating Intervention Characteristics).

**B. Policies, strategies, and practices for vaccinating adolescent girls with HIV**

1. Specifically, for adolescent girls with HIV, what are the strategies implemented to vaccinate this cohort? (PRISM: Evaluating Intervention Characteristics)
   1. Probe on strategies for specifically identifying and vaccinating HIV+ and immunocompromised girls vs general population. Are HPV vaccines delivered in ART clinics? If yes, probe on who is responsible and how vaccines are received and stored.
   2. Since Uganda will be adopting the single dose schedule, probe for the approach to completely vaccinate HIV+ and immunocompromised girls (adolescent girls with HIV) if different.
   3. Could you please provide any policy documents or guidelines that detail the strategies for vaccinating adolescent girls with HPV, particularly those who are HIV-positive or immunocompromised?
2. To your knoweldge, are there any specific programs or initiatives aimed at improving HPV vaccination rates among HIV+ and immunocompromised girls (adolescent girls with HIV)? If so, can you describe them? (PRISM: Assessing External Environment)
3. In your opinion, are there any promising practices for HPV vaccination for adolescent girls with HIV that you are aware of in Uganda? (PRISM: Assessing External Environment)

C**. Assessing perception (awareness, feasibility, and acceptability) of vaccinating HIV+ and immunocompromised girls (adolescent girls with HIV)**

1. In your words, briefly explain why HPV vaccination is especially important for adolescents living with HIV

From your perspective, how would you rate the sufficiency of the current strategies towards reaching HIV+ and immunocompromised girls with HPV vaccines. Please rate from 1-5 to rank sufficiency with 1- Not sufficient at all, 2 - Not sufficient, 3 -Neutral 4- Somewhat sufficient, 5 - Very/completely sufficient (PRISM: Assessing External Environment)

In your opinion, briefly explain the factors influencing HPV vaccination in HIV+ and immunocompromised girls (adolescent girls with HIV). (PRISM: Assessing External Environment)

1. Have you noticed any changes in the attitudes and perceptions of different stakeholders towards the HPV vaccination for HIV+ and immunocompromised girls (adolescent girls with HIV)?

3. Are there other initiatives beyond those mentioned above that influenced the acceptability and uptake of the HPV vaccines in HIV+ and immunocompromised girls? Kindly elaborate (PRISM: Assessing External Environment)

1. From your perspective, how would you rate the feasibility of implementing effective strategies for reaching HIV+ and immunocompromised girls with HPV vaccines. Please rate from 1-5 to rank feasibility with 1- Not feasible at all, 2 - Not feasible, 3 -Neutral 4- Somewhat feasible, 5 - Very/completely feasible (PRISM: Evaluating Intervention Characteristics)

D. **Barriers and facilitators to HPV vaccination** **in HIV+ and immunocompromised girls (adolescent girls with HIV)**

1. From your perspective, what are the main challenges EPI/healthcare providers face in vaccinating HIV+ and immunocompromised girls (adolescent girls with HIV) against HPV?
   1. Probe: How do logistical issues, such as vaccine availability and access to healthcare facilities, affect HPV vaccination rates among HIV+ and immunocompromised girls (adolescent girls with HIV)? (PRISM: Assessing External Environment)
2. In your opinion, can you describe any misconceptions or fears about the HPV vaccine amongst parents or guardians of HIV+ and immunocompromised girls (adolescent girls with HIV)? (PRISM: Understanding Recipient Characteristics)
3. In your opinion, are there gender-specific, social or cultural norms that influence ALHIV vaccination? (PRISM: Assessing External Environment)
4. How do logistical issues, such as vaccine availability and access to healthcare facilities, affect HPV vaccination rates among HIV+ and immunocompromised girls (adolescent girls with HIV)? (PRISM: Assessing External Environment)
5. Are there any specific factors that have facilitated the vaccination of HIV+ and immunocompromised girls (adolescent girls with HIV) with the HPV vaccine in your setting? (PRISM: Assessing External Environment)
   1. Probe: if yes, please explain these factors
   2. Are there any programs that enabled the vaccination of HIV+ and immunocompromised girls (adolescent girls with HIV)?
6. In your opinion, what are some of the best practices or successful strategies that have been reported so far from the field in the implementation of HPV vaccination in HIV+ and immunocompromised girls (adolescent girls with HIV)? (PRISM: Assessing External Environment)
   1. Probe: Can you share some specific examples of successful interventions or initiatives that have positively influenced the acceptability and uptake of the HPV vaccine in HIV+ and immunocompromised girls (adolescent girls with HIV)?

**E. Assessing opportunities for better integration of HPV vaccination for HIV+ and immunocompromised girls (adolescent girls with HIV) at scale.**

1. Based on your insights and experience, what other recommendations would you make to sustain and further increase HPV vaccination coverage among HIV+ and immunocompromised girls (adolescent girls with HIV)? Probe on strategies for reaching through facility, school and community strategies (PRISM: Assessing implementation and sustainability infrastructure)

a. Are there any additional insights or recommendations you would like to share regarding HPV vaccination in HIV+ and immunocompromised girls (adolescent girls with HIV)?

# Uganda

## Key Informant Interview Guide

**Stakeholder group:** National and Subnational level stakeholders

**Interviewer:** Thank you for agreeing to participate in this interview. As a reminder, this interview should take approximately 30-45 minutes to complete, and there are no right or wrong answers. We would like your insights on the implementation of HPV vaccination policies for People Living with HIV (PLHIV) especially HIV+ and immunocompromised girls (also known as adolescent girls with HIV). These interviews will be conducted with stakeholders, like yourself who play a key role at the national or subnational level in the HPV vaccination program.

If you agree, your interview will be audio-recorded and stored on a secure server. No individual data will be released or made public in any way. Your responses will remain confidential, and your name will not be associated with your responses. You do not have to answer every question, and you can end the interview at any time. Your participation in this interview is completely voluntary. Is it ok if we audio record?

Do you have any questions before we start? [Answer questions.]

The interviewer administers the consent form and the interviewee signs before the interview starts.

KII Interview questions:

**Organization: _____________________________**

**Designation of Stakeholder: ____________________**

**Age: ___________________**

**Gender: _________________**

**Number of years in healthcare service:**

**Date: ____ /____ /____**

**Identifier code:**

**A. General Knowledge on HPV vaccination:** *The goal of this study is to learn about the policies and strategies that may help improve HPV vaccination completion for HIV + and immunicompromised gilrs in Uganda. We want to learn from your experience that you have with either general or HPV vaccine specific strategies that might work to improve HPV vaccination completion for HIV + and immunicompromised gilrs in Uganda.*

1. When was HPV vaccine introduced in your country? Month and year

(**Stakeholders:** Ministry of Health, EPI Manager, Adolescent Health Manager, Partners Supporting the HPV Program, District Health Officer, Assistant District Health Officer-Marternal Child Health, HIV Focal Person and other stakeholders who are extensively involved in HPV vaccination perspectives on the HPV vaccination)

1. What was your role during the HPV vaccine introduction?

(**Stakeholders:** Ministry of Health, EPI Manager, Adolescent Health Manager, Partners Supporting the HPV Program, Ministry of Gender and Labour, Ministry of Education and Sports District Health Officer, Assistant District Health Officer-Marternal Child Health, HIV Focal Person and other stakeholders who are extensively involved in HPV vaccination perspectives on the HPV vaccination)

1. How many doses of HPV vaccines are given to adolescent girls?

(**Stakeholders:** Ministry of Health, EPI Manager, Adolescent Health Manager, Partners Supporting the HPV Program, District Health Officer, Assistant District Health Officer-Marternal Child Health, HIV Focal Person and other stakeholders who are extensively involved in HPV vaccination perspectives on the HPV vaccination)

1. How many doses are given to adolescents girls living with HIV? Uganda is adopting the single dose schedule and the switch is yet to happen, probe if the guidelines for doses to adolescent girls living with HIV will change with the adoption of single dose HPV schedule, perhaps from 3 dose to 2 dose? (PRISM: Evaluating Intervention Characteristics) (**Stakeholders:** Ministry of Health, EPI Manager, Adolescent Health Manager, Partners Supporting the HPV Program, District Health Officer, Assistant District Health Officer-Marternal Child Health, HIV Focal Person and other stakeholders who are extensively involved in HPV vaccination perspectives on the HPV vaccination)
2. Since HPV vaccine introduction, what strategies have been implemented to vaccinate adolescent girls? Probe on school based, facility based and community based strategies. (PRISM: Evaluating Intervention Characteristics) (**Stakeholders:** Ministry of Health, EPI Manager, Adolescent Health Manager, Partners Supporting the HPV Program, Ministry of Education, Gender and Labour, Ministry of Education and Sports, District Health Officer, Assistant District Health Officer-Marternal Child Health, HIV Focal Person and other stakeholders who are extensively involved in HPV vaccination perspectives on the HPV vaccination. )

**B. Policies, strategies, and practices for vaccinating adolescent girls with HIV**

1. Specifically, for adolescent girls with HIV, what are the strategies implemented to vaccinate this cohort? (PRISM: Evaluating Intervention Characteristics)
   1. Probe on strategies for specifically identifying and vaccinating HIV+ and immunocompromised girls vs general population. Are HPV vaccines delivered in ART clinics? If yes, probe on who is responsible and how vaccines are received and stored.
   2. Since Uganda will be adopting the single dose schedule, probe for the approach to completely vaccinate HIV+ and immunocompromised girls (adolescent girls with HIV) if different.
   3. Could you please provide any policy documents or guidelines that detail the strategies for vaccinating adolescent girls with HPV, particularly those who are HIV-positive or immunocompromised?
2. To your knoweldge, are there any specific programs or initiatives aimed at improving HPV vaccination rates among HIV+ and immunocompromised girls (adolescent girls with HIV)? If so, can you describe them? (PRISM: Assessing External Environment)
3. In your opinion, are there any promising practices for HPV vaccination for adolescent girls with HIV that you are aware of in Uganda? (PRISM: Assessing External Environment)

C**. Assessing perception (awareness, feasibility, and acceptability) of vaccinating HIV+ and immunocompromised girls (adolescent girls with HIV)**

1. In your words, briefly explain why HPV vaccination is especially important for adolescents living with HIV

From your perspective, how would you rate the sufficiency of the current strategies towards reaching HIV+ and immunocompromised girls with HPV vaccines. Please rate from 1-5 to rank sufficiency with 1- Not sufficient at all, 2 - Not sufficient, 3 -Neutral 4- Somewhat sufficient, 5 - Very/completely sufficient (PRISM: Assessing External Environment)

In your opinion, briefly explain the factors influencing HPV vaccination in HIV+ and immunocompromised girls (adolescent girls with HIV). (PRISM: Assessing External Environment)

1. Have you noticed any changes in the attitudes and perceptions of different stakeholders towards the HPV vaccination for HIV+ and immunocompromised girls (adolescent girls with HIV)?
2. Are there other initiatives beyond those mentioned above that influenced the acceptability and uptake of the HPV vaccines in HIV+ and immunocompromised girls? Kindly elaborate (PRISM: Assessing External Environment)
3. From your perspective, how would you rate the feasibility of implementing effective strategies for reaching HIV+ and immunocompromised girls with HPV vaccines. Please rate from 1-5 to rank feasibility with 1- Not feasible at all, 2 - Not feasible, 3 -Neutral 4- Somewhat feasible, 5 - Very/completely feasible (PRISM: Evaluating Intervention Characteristics)

D. **Barriers and facilitators to HPV vaccination** **in HIV+ and immunocompromised girls (adolescent girls with HIV)**

1. From your perspective, what are the main challenges EPI/healthcare providers face in vaccinating HIV+ and immunocompromised girls (adolescent girls with HIV) against HPV?
   1. Probe: How do logistical issues, such as vaccine availability and access to healthcare facilities, affect HPV vaccination rates among HIV+ and immunocompromised girls (adolescent girls with HIV)? (PRISM: Assessing External Environment)
2. In your opinion, can you describe any misconceptions or fears about the HPV vaccine amongst parents or guardians of HIV+ and immunocompromised girls (adolescent girls with HIV)? (PRISM: Understanding Recipient Characteristics)
3. In your opinion, are there gender-specific, social or cultural norms that influence ALHIV vaccination? (PRISM: Assessing External Environment)
4. How do logistical issues, such as vaccine availability and access to healthcare facilities, affect HPV vaccination rates among HIV+ and immunocompromised girls (adolescent girls with HIV)? (PRISM: Assessing External Environment)
5. Are there any specific factors that have facilitated the vaccination of HIV+ and immunocompromised girls (adolescent girls with HIV) with the HPV vaccine in your setting? (PRISM: Assessing External Environment)
   1. Probe: if yes, please explain these factors
   2. Are there any programs that enabled the vaccination of HIV+ and immunocompromised girls (adolescent girls with HIV)?
6. In your opinion, what are some of the best practices or successful strategies that have been reported so far from the field in the implementation of HPV vaccination in HIV+ and immunocompromised girls (adolescent girls with HIV)? (PRISM: Assessing External Environment)
   1. Probe: Can you share some specific examples of successful interventions or initiatives that have positively influenced the acceptability and uptake of the HPV vaccine in HIV+ and immunocompromised girls (adolescent girls with HIV)?

**E. Assessing opportunities for better integration of HPV vaccination for HIV+ and immunocompromised girls (adolescent girls with HIV) at scale.**

1. Based on your insights and experience, what other recommendations would you make to sustain and further increase HPV vaccination coverage among HIV+ and immunocompromised girls (adolescent girls with HIV)? Probe on strategies for reaching through facility, school and community strategies (PRISM: Assessing implementation and sustainability infrastructure)

a. Are there any additional insights or recommendations you would like to share regarding HPV vaccination in HIV+ and immunocompromised girls (adolescent girls with HIV)?

## Focus group discussion Guide

**Date: ____ /____ /____**

| **Designation of HCW** | **Gender** | **Name of Facility** | **Setting (urban/rural)** | **Identifier code** |
| --- | --- | --- | --- | --- |
|  |  |  |  |  |
|  |  |  |  |  |
|  |  |  |  |  |
|  |  |  |  |  |
|  |  |  |  |  |
|  |  |  |  |  |
|  |  |  |  |  |
|  |  |  |  |  |
|  |  |  |  |  |

**A. General Knowledge on HPV vaccination**

1. Can anyone share what they know about HPV vaccination? And the diseases that HPV vaccine can protect against? How it might be different for girls with HIV? (PRISM: Evaluating Intervention Characteristics)
2. When was HPV vaccine introduced in your facility? Month and year (PRISM: Evaluating Intervention Characteristics)
3. How many doses of HPV vaccines are given to adolescent girls? (PRISM: Evaluating Intervention Characteristics)
4. How many doses of HPV vaccines are given to HIV+ and immunocompromised girls (adolescent girls with HIV)? Since Uganda will be adopting a single dose schedule, probe if the guidelines for doses to adolescent girls living with HIV will change with the adoption of single dose HPV schedule, perhaps from 3 dose to 2 dose? (PRISM: Evaluating Intervention Characteristics)
5. Since HPV vaccine introduction, what policy documents are vailable and or strategies that have been implemented to vaccinate adolescent girls? Probe on school based, facility based and community based strategies (PRISM: Evaluating Intervention Characteristics)

**B. Policies, strategies and practices for vaccinating HIV+ and immunocompromised girls (adolescent girls with HIV)**

1. Can anyone share the policies and strategies for vaccinating HIV+ and immunocompromised girls (adolescent girls with HIV)? (PRISM: Evaluating Intervention Characteristics)
   1. Probe on policies and strategies for specifcially identifying and vaccinating HIV+ and immunocompromised girls vs general population. Are HPV vaccines delivered in ART clinics? If yes, probe on who is responsible and how vaccines are received and stored (PRISM: Evaluating Intervention Characteristics)
   2. Since the Uganda will be adopting the single dose schedule, probe for the approach to completely vaccinate HIV+ and immunocompromised girls (adolescent girls with HIV) if different. (PRISM: Evaluating Intervention Characteristics)
2. To your knowledge, are there any specific programs or initiatives aimed at improving HPV vaccination rates among HIV + and immunocompromised girls? If so, can you describe them? (PRISM: Assessing External Environment)
3. Are there any promising practices for HPV vaccination for adolescent girls with HIV that you are aware of inUganda? If so, can you describe them? (PRISM: Assessing External Environment)

C**. Assessing perception (awareness, feasibility, and acceptability) of vaccinating HIV+ and immunocompromised girls (adolescent girls with HIV)**

1. In your words, briefly explain why HPV vaccination is especially important for adolescents living with HIV
2. In your opinion, briefly explain the factors influencing HPV vaccination in HIV+ and immunocompromised girls (adolescent girls with HIV)?
3. How do different stakeholders perceive HPV vaccination in ALHIV?
   1. Probe: Are there differences among women vs men, healthcare workers vs school admin/teachers vs parents vs others?
4. Are there other initiatives beyond those mentioned above that influenced the acceptability and uptake of the HPV vaccines in HIV+ and immunocompromised girls? Kindly elaborate (PRISM: Assessing External Environment)

D. **Barriers and facilitators to HPV vaccination in HIV+ and immunocompromised girls (adolescent girls with HIV)**

1. From your perspective, what are the main challenges EPI/healthcare providers face in vaccinating adolescent girls against HPV?
   1. Probe: How do logistical issues, such as vaccine availability and access to healthcare facilities, affect HPV vaccination rates among adolescent girls?
2. In your opinion, can you describe any misconceptions or fears about the HPV vaccine amongst parents or guardians of ALHIV? (PRISM: Assessing External Environment)
3. In your opinion, are there gender-specific, social or cultural norms that influence ALHIV vaccination? (PRISM: Assessing External Environment)
4. How do logistical issues, such as vaccine availability and access to healthcare facilities, affect HPV vaccination rates among ALHIV girls? (PRISM: Assessing External Environment)
5. How do HIV related issues, such as issues related to navigating the complexities of managing both disclosure and HIV treatment, stigma, including internalized stigma, and discrimination, affect HPV vaccination rates among ALHIV girls? (PRISM: Assessing External Environment)
6. Are there any specific factors that have encouraged parents/caregivers or adolescents to bring their child or self to the facility for vaccination? (PRISM: Assessing External Environment)
   1. Probe: if yes, please explain these factors
   2. Are there any programs that enabled the vaccination of HIV+ and immunocompromised girls (adolescent girls with HIV)?
   3. Are there any communication or social mobilization strategies that promoted awareness about HPV vaccination in HIV+ and immunocompromised girls (adolescent girls with HIV)?
7. In your opinion, what are some of the best practices or successful strategies that have been reported so far from the field in the implementation of HPV vaccination in HIV+ and immunocompromised girls (adolescent girls with HIV)? (PRISM: Assessing implementation and sustainability infrastructure)
   1. Probe: Can you share some specific examples of successful interventions or initiatives that have positively influenced the acceptability and uptake of the HPV vaccine in HIV+ and immunocompromised girls (adolescent girls with HIV)?

**E. Assessing opportunities for better integration of HPV vaccination for HIV+ and immunocompromised girls (adolescent girls with HIV) at scale.**

1. Based on your insights and experience, what recommendations would you make to sustain and further increase HPV vaccination coverage among HIV+ and immunocompromised girls (adolescent girls with HIV) in Uganda?
2. Is there any other thing anyone wants to share regarding HPV vaccination in HIV+ and immunocompromised girls (adolescent girls with HIV)?
