# Supplementary material for: Policies and strategies for HPV vaccination schedule completion in immunocompromised girls, including girls living with HIV: Qualitative insights from Eswatini, Malawi, and Uganda
Source: PLOS Glob Public Health. 2026 Feb 3;6(2):e0004688. doi: 10.1371/journal.pgph.0004688 (PMC12867219; doi:10.1371/journal.pgph.0004688)
Supplement: Attachment — Submitted filename: Editors Report_HPV vaccination among girls living with HIV.pdf [file pgph.0004688.s005.pdf]

Report

Policies and strategies for HPV vaccination schedule completion in immunocompromised girls, including girls living with HIV: Qualitative insights from Eswatini, Malawi, and Uganda

<u>Editor's General Comments</u>

Thank you for the opportunity to review this manuscript. The authors report on a pivotal research project aimed at exploring and understanding gaps in HPV immunization policy and practice for the most at-risk population, adolescent girls living with HIV. The findings are telling, highlight how health systems within high burden countries are ill-equipped and poorly designed to meet the HPV vaccination needs of this population. This raises critical concerns for how to enhance equitable access to cervical cancer prevention services for adolescent girls living with HIV. While the research focus aligns with a critical public health concern, I have some major and minor comments including some reservations in terms of how the methods have been reported and how the analysis was conducted in this study.

Major Comments:

While the introduction section adequately appraises evidence on the disproportionate burden of HPV and cervical cancer among women living with HIV in the African region, it fails to provide sufficient context on the performance of cervical cancer prevention strategies (including HPV vaccination programs) within the African region compared to elsewhere, including implementation, coverage, uptake and acceptance.

The sub-section in the introduction on the safety and efficacy of HPV vaccines among immunosuppressed individuals will benefit from discussing ongoing clinical trials exploring the immunogenicity of single dose vaccination within this population and the potential policy implications of the anticipated findings.

The authors' description of the desk review in the methods section is unfortunately insufficient. The manuscript may benefit from a dedicated paragraph/sub-section unpacking the approach used to collect and analyse data from the desk review component of the study. As it stands, it is unclear from the methods section how sources were identified (e.g., eligibility criteria for selection and inclusion in the study), how data was extracted from eligible sources or how they were analysed alongside data from the qualitative study component.

Authors should provide sound justification or rationale for using ChatGPT for the purpose of thematic analysis, say, as opposed to established qualitative analysis tools/software. Further to this, the authors should comment on the analytical approach / coding used to prompt ChatGPT and the analytical framework or theory within which the analysis was grounded in order to enhance transparency and reproducibility. This includes expanding on the "thematic sections" and how these were derived. Where any limitations exit in their use of ChatGPT for the purpose of the thematic analysis, these should be fully acknowledged and substantiated in the limitations section of the manuscript.

It is typically common practice in qualitative research studies that findings are reported alongside excerpts from transcripts. Unfortunately, the authors have not provided any quotes from participants of the KII or FGDs. This makes it challenging to fully appreciate the experiences of participants across the stakeholders and countries studied.

Further to the core areas examined in this study, it would have been important to understand if national health plans factor-in the additional dose(s) required by girls living with HIV or any other form of immunodeficiency in line with the national policies. For example, does the country make a financial allocation to cover the programmatic costs (besides vaccine procurement costs) associated with delivering additional doses to this population outside of the national routine structures. The authors should comment on whether this was explored and the implications thereof.

As part of the limitations of the study, the authors suggest that the use of qualitative methods limits generalizability of the findings. I am not convinced that the qualitative research approach inherently introduces limitations in terms of the generalizability of findings. The authors should critically examine the study design, data collection methods and analytical approaches in informing the specific limitations and biases introduced into the study.

Minor Comments:

- Lines 79 onwards: Authors should indicate ages in years throughout the document for ease of clarity
- Line 118: "*...the region, with age-standardized incidence 67.9 and 56.2 per 100,000, respectively...*" Authors should review this sentence for clarity
- Line 157: "*The guides were developed using...*" The authors should provide templates of the guides used as part of supplementary materials for ease of reference. Further to this, the authors should clarify if (and how) the guides were pretested or piloted and document any amendments made following this
- Line 165: "*All data was analyzed by thematic analysis ...*" Replace "was" with "were"
- Lines 167-168: "*Raw data was shared with ChatGPT...*" By raw data, are the authors referring to audio recordings or transcripts of the audio recordings. Replace "was" with "were"
- Line 207: Table 2: The year for which coverage has been reported should be indicated for ease of reference
- Line 243: "*Policy, documentation, and awareness about vaccinating immunocompromised girls...*" Authors do not specify or cite the specific sources included in the document review for this study.
- Lines 247-248: "*...the main delivery strategy for girls with HIV was delivering the HPV vaccine to girls with HIV through...*" The authors should kindly review the sentence and rephrase for clarity. The authors should note the inconsistent use of the abbreviation GLHIV as it is sometimes written out in full as in this sentence. This applies to all other abbreviations used in the manuscript
- Lines 305-307: It is unclear if this is deduced from the qualitative component or the desk review, or a discussion of the findings (in which case, any interpretation or appraisal of the results should be left for the discussion section)
- Lines 315-316: "*...as well as sporadic meeting dates and transportation challenges for GLHIV...*" The "sporadic meeting dates" referred to is unclear in the context of the study. The authors may want to unpack this further to enhance clarity on this finding
- Lines 474-475: "*Another important enabler for HIV vaccination across the three countries...*" Do the authors mean "HPV vaccination" and not "HIV vaccination"?
