# Supplementary material for: Policies and strategies for HPV vaccination schedule completion in immunocompromised girls, including girls living with HIV: Qualitative insights from Eswatini, Malawi, and Uganda
Source: PLOS Glob Public Health. 2026 Feb 3;6(2):e0004688. doi: 10.1371/journal.pgph.0004688 (PMC12867219; doi:10.1371/journal.pgph.0004688)
Supplement: Attachment — Submitted filename: REVIEWER_COMMENTS_23.07.25.docx [file pgph.0004688.s004.docx]

**Policies and strategies for HPV vaccination schedule completion in immunocompromised girls, including girls living with HIV: Qualitative insights from Eswatini, Malawi, and Uganda.**

This study is highly policy relevant. Please consider doing justice to such a study by improving sentence clarity, tightening your arguments, reporting/synthesizing the results better and making the discussion more analytical rather than descriptive.

**Introduction**

Your introduction is **comprehensive and well-referenced**, but it can be significantly improved by addressing several structural, stylistic, and logical issues.

Important arguments (e.g., the syndemic of HIV and HPV) are **buried midway** rather than foregrounded.

The logic leading from the **global problem** to the **regional context** and finally to the **study focus** could be clearer.

Several facts are repeated unnecessarily (e.g., HPV causes cervical cancer is stated more than once).

The core **research problem** (the lack of intentional HPV vaccine delivery for girls living with HIV) is only introduced at the end (line 135), but should appear much earlier.

There’s a lack of clear **topic sentences** for each paragraph

I would recommend you synthesize what you have into these subtitles;

**Background**

- Burden of cervical cancer globally and in LMICs
- Relationship between HPV, HIV, and cervical cancer

**Vulnerability of WLHIV and GLHIV**

- Increased risk and worse outcomes
- Biological + structural vulnerabilities

**Global Policy Landscape**

- WHO 90-70-90 targets
- Vaccine guidelines for immunocompromised populations

**Regional Focus: SSA (Eswatini, Malawi, Uganda)**

- Syndemic of HIV and HPV
- Specific stats for GLHIV and regional incidence/mortality

**Gap and Study Rationale**

- Evidence gap on tailored implementation for GLHIV
- Hypothesis and study objective

Line 130-132; You say that evidence is limited but don’t mention how. I would demonstrate the evidence limitations to back up that claim.

It would be useful to summarize the HIV and cervical cancer stats (ASIR, ASMR, projections by 2045) for all 3 countries in a Table

What is the relevance of Lines 58-59; 75-76; 92-93; 107-108

**Methods**

Line 139; What is a cross-sectional qualitative approach?

Line 140; Who are these stakeholders? Give specifics and justification for picking them

Please clarify how sampling was conducted.

The desk review is described, but it's unclear **how findings were integrated** into the analysis (e.g., were they analyzed thematically too?).

Line 145; What is this “other grey literature”

Lines 148-152; this paragraph is better situated as the last part of the methods section

Clearly state **how ChatGPT was used** (e.g., was it prompted with summaries, full transcripts, or coded excerpts?). Clarify whether outputs were used **exploratorily or as part of the primary coding process**.

Thematic analysis approach is underspecified; No mention of whether **inductive or deductive coding** was used. No mention of **steps followed** (e.g., familiarization, coding, theme generation).

In the current **methods section, there is no mention of trustworthiness**, which is a crucial component in qualitative research to demonstrate **credibility, dependability, confirmability, and transferability** of findings.

**Results**

Table 1; You speak of partners and health care workers. I asked for this to be better defined in the methods section

Are there any verbatim quotes you can add to your results section to boost their credibility? It is a qualitative study after all

**This section is overly descriptive, under-analytical.** Much of the text reads like field notes or summaries, rather than synthesized findings. Please adjust

Line 186-206;This section feels like background or methods, not results. No integration with interview/FGD data.

Line 208-224; almost no attribution to interview data. missing statements like “Respondents in X country noted...”

Line 225-241; Needs more structure. Combine this with the policy section as “Policy and Awareness.”

Line 242-316; Too repetitive. structure is based on countries instead of emergent themes. Long paragraphs with weak transitions and little synthesis.

Results could be strengthened by exploring differences by stakeholder type (e.g., government vs NGO vs health worker), or region/district.

Thematic analysis was cited in methods, but the results are not presented as such.

**Discussion**

The discussion is not anchored in any conceptual or theoretical model (e.g., health systems strengthening, implementation science, socio-ecological models).

**Consider** Use a framework like the WHO building blocks or PRISM (used in the methods) to interpret findings and propose recommendations.

Line 405; Novel insights? Really?

The first paragraph of the discussion ought to recap study objectives and summarize key findings. Please adjust.

Line 411; Not the place for this. Also this is what did not come out clearly in the gap analysis in the introduction

Consider doing away with the subheadings in the discussion. Just discuss one idea per paragraph

Line 413; This paragraph is descriptive, not analytical and fails to ask why such gaps persist. Misses opportunity to connect to literature on policy implementation failures in LMICs. Discuss power dynamics, health system fragmentation, and siloed programming (e.g., EPI vs HIV programs). Integrate literature on implementation science or health systems bottlenecks.

Line 438-450; Does not distinguish between general HPV barriers and those unique to GLHIV. No analysis of underlying causes (e.g., funding, capacity, stigma).

Line 451-464; No critical reflection on how to address these issues, especially among GLHIV. Use this section to reflect on how stigma intersects with gender and age. Consider adding a brief reflection on community engagement strategies that have worked elsewhere (e.g., peer educators, youth-led advocacy).

Line 465-476; Use synthesis: rather than listing what exists, explain how and why these mechanisms are effective. Anchor enablers within a theory (e.g., trust, continuity of care, peer norms).

Line 477-479; Lacks critical appraisal of feasibility or sustainability. Reflect on whether such strategies are scalable and equitable. Mention contextual adaptation; what works in Eswatini may not work in Malawi.

**Limitations**

This section can be synthesized better. Also add study strengths

**Policy implications**

Some paragraphs are long and could be broken down for clarity. Certain sentences are also dense with multiple ideas.

Some ideas are repeated or could be collapsed (e.g., several mentions of ART clinics and Teen Clubs across different paragraphs).

Use more measured academic language. Replace “Countries should…” with “National programs may consider…” or “Evidence suggests…”

No mention of **health system constraints** (financing, HR, competing priorities) that may hinder implementation.

The section sometimes reads like a general policy proposal. It needs stronger anchoring in study findings. Use phrasing like:

- “As evidenced by stakeholder feedback in Eswatini...”
- “Our results showed that in Uganda…”
- “Health worker perspectives highlighted…”
