# Supplementary material for: Policies and strategies for HPV vaccination schedule completion in immunocompromised girls, including girls living with HIV: Qualitative insights from Eswatini, Malawi, and Uganda
Source: PLOS Glob Public Health. 2026 Feb 3;6(2):e0004688. doi: 10.1371/journal.pgph.0004688 (PMC12867219; doi:10.1371/journal.pgph.0004688)
Supplement: Attachment — Submitted filename: HAPPI Manuscript Response to Reviewers 22SEP2025.docx [file pgph.0004688.s007.docx]

Dear Editor and Reviewer,

We are pleased to share the revised manuscript for the paper titled “Policies and strategies for HPV vaccination schedule completion in immunocompromised girls, including girls living with HIV: Qualitative insights from Eswatini, Malawi, and Uganda,” which we submitted for review earlier this year. The comments we received were comprehensive and constructive, and we are grateful for the attention and level of detail in the guidance received. Based on these comments, we have made major revisions to the manuscript.

In response to the comments received, we have restructured the manuscript outline, paying special attention to the structure and coherence of the introduction and discussion sections. The methods section has been updated to provide more detail, and we now include the interview guides as supplemental materials. The results section has been revised to be more concrete and synthesized, and the focus is now more clearly aligned with thematic results rather than country results. We have also included quotes from the recordings to support our findings. The discussion section has been updated to more clearly align with the PRISM framework that guided the data collection tools, and the policy implications section has been revised to carry more comprehensive considerations and a more appropriate tone.

Our detailed responses to the comments follow this letter.

Thank you for considering this resubmission. We believe that this manuscript is much stronger thanks to the comments received, and remain available to discuss or address any further comments or questions.

Sincerely,
**Sonali M. Patel**
On behalf of all co-authors
[*spatel@clintonhealthaccess.org*](mailto:spatel@clintonhealthaccess.org)
Global Vaccines Delivery Team, Clinton Health Access Initiative

*Editor comments*

| While the introduction section adequately appraises evidence on the disproportionate burden of HPV and cervical cancer among women living with HIV in the African region, it fails to provide sufficient context on the performance of cervical cancer prevention strategies (including HPV vaccination programs) within the African region compared to elsewhere, including implementation, coverage, uptake and acceptance. | We have addressed this in lines 138-140 with the text “Primary prevention of cervical cancer is particularly important in these countries as access to cervical cancer prevention and treatment remains limited [31] while cervical cancer prevalence is increasing [32].” |
| --- | --- |
| The sub-section in the introduction on the safety and efficacy of HPV vaccines among immunosuppressed individuals will benefit from discussing ongoing clinical trials exploring the immunogenicity of single dose vaccination within this population and the potential policy implications of the anticipated findings. | Reference to these trials has been added. (Lines 127-129): “. Although ongoing clinical trials to evaluate the efficacy of a single-dose HPV vaccine in HIV-positive individuals may affect future vaccine recommendations [28,29], a differentiated dose schedule for GLHIV and other immunocompromised girls is currently considered essential” |
| The authors’ description of the desk review in the methods section is unfortunately insufficient. The manuscript may benefit from a dedicated paragraph/sub-section unpacking the approach used to collect and analyse data from the desk review component of the study. As it stands, it is unclear from the methods section how sources were identified (e.g., eligibility criteria for selection and inclusion in the study), how data was extracted from eligible sources or how they were analysed alongside data from the qualitative study component. | These questions have been addressed in a new paragraph describing the desk review (lines 170-180), as well as inclusion of a table in the results explaining which documents were obtained in the desk review (Table 3 Summary of documents identified for the desk review). |
| Authors should provide sound justification or rationale for using ChatGPT for the purpose of thematic analysis, say, as opposed to established qualitative analysis tools/software. Further to this, the authors should comment on the analytical approach / coding used to prompt ChatGPT and the analytical framework or theory within which the analysis was grounded in order to enhance transparency and reproducibility. This includes expanding on the "thematic sections" and how these were derived. Where any limitations exit in their use of ChatGPT for the purpose of the thematic analysis, these should be fully acknowledged and substantiated in the limitations section of the manuscript. | This has been addressed in the methods section (lines 203-210) as well as the limitations section (549-551). |
| It is typically common practice in qualitative research studies that findings are reported alongside excerpts from transcripts. Unfortunately, the authors have not provided any quotes from participants of the KII or FGDs. This makes it challenging to fully appreciate the experiences of participants across the stakeholders and countries studied. 1 | We have updated the results section to include quotes to support the findings. |
| Further to the core areas examined in this study, it would have been important to understand if national health plans factor-in the additional dose(s) required by girls living with HIV or any other form of immunodeficiency in line with the national policies. For example, does the country make a financial allocation to cover the programmatic costs (besides vaccine procurement costs) associated with delivering additional doses to this population outside of the national routine structures. The authors should comment on whether this was explored and the implications thereof. | This has been addressed in the limitations section, lines 541-543:  “The study design did not include a review of program budgets or financial records, which limits our ability to draw conclusions about the role of resource constraints in vaccinating GLHIV.” |
| As part of the limitations of the study, the authors suggest that the use of qualitative methods limits generalizability of the findings. I am not convinced that the qualitative research approach inherently introduces limitations in terms of the generalizability of findings. The authors should critically examine the study design, data collection methods and analytical approaches in informing the specific limitations and biases introduced into the study. | This section has been revised and expanded to include more relevant limitations. |

| • Lines 79 onwards: Authors should indicate ages in years throughout the document for ease of clarity | The manuscript now includes “years” with all reference to ages |
| --- | --- |
| • Line 118: “…the region, with age-standardized incidence 67.9 and 56.2 per 100,000, respectively…” Authors should review this sentence for clarity | This has been revised for clarity to read: “Eswatini, Malawi, and Uganda are key countries for cervical cancer prevention; cervical cancer incidence per 100,000 women is 84.6 in Eswatini, 67.9 in Malawi, and 56.2 in Uganda” |
| • Line 157: “The guides were developed using…” The authors should provide templates of the guides used as part of supplementary materials for ease of reference. Further to this, the authors should clarify if (and how) the guides were pretested or piloted and document any amendments made following this | We have added a sentence on the piloting the guides (line 187, “Guides were piloted in each country and adjusted for clarity.” and include the guides as supplementary materials (S1 Interview Guides). |
| • Line 165: “All data was analyzed by thematic analysis …” Replace "was" with "were" | This has been revised |
| • Lines 167-168: “Raw data was shared with ChatGPT…” By raw data, are the authors referring to audio recordings or transcripts of the audio recordings. Replace "was" with "were" | This has been updated for clarity and now reads “Data from the note-taking matrices was uploaded to ChatGPT, which was prompted to conduct a thematic analysis of the qualitative data.” |
| • Line 207: Table 2: The year for which coverage has been reported should be indicated for ease of reference | The row label has been revised to read “HPV1 coverage (2023) “ |
| • Line 243: “Policy, documentation, and awareness about vaccinating immunocompromised girls…” Authors do not specify or cite the specific sources included in the document review for this study. | This has been addressed with the inclusion of Table 3. |
| • Lines 247-248: “…the main delivery strategy for girls with HIV was delivering the HPV vaccine to girls with HIV through…” The authors should kindly review the sentence and rephrase for clarity. The authors should note the inconsistent use of the abbreviation GLHIV as it is sometimes written out in full as in this sentence. This applies to all other abbreviations used in the manuscript | This has been updated throughout the document |
| • Lines 305-307: It is unclear if this is deduced from the qualitative component or the desk review, or a discussion of the findings (in which case, any interpretation or appraisal of the results should be left for the discussion section) | This has been revised to clearly state that the information comes from respondents |
| • Lines 315-316: “…as well as sporadic meeting dates and transportation challenges for GLHIV…” The "sporadic meeting dates" referred to is unclear in the context of the study. The authors may want to unpack this further to enhance clarity on this finding | This language has been updated for clarity and now reads “Challenges to delivery through Teen Clubs were also noted, namely a lack of vaccine cold storage in meeting venues, irregular Teen Club meeting dates, and a lack of reliable transportation for some GLHIV to attend meetings” |
| • Lines 474-475: “Another important enabler for HIV vaccination across the three countries…” Do the authors mean "HPV vaccination" and not "HIV vaccination"? | This has been corrected to read “HPV vaccination” |

*Reviewer comments*

| Reviewer comment | Status |
| --- | --- |
| This study is highly policy relevant. Please consider doing justice to such a study by improving sentence clarity, tightening your arguments, reporting/synthesizing the results better and making the discussion more analytical rather than descriptive. | Thank you for this assessment, we have conducted a comprehensive revision of the manuscript with the aim of addressing this and other comments. |
| Your introduction is **comprehensive and well-referenced**, but it can be significantly improved by addressing several structural, stylistic, and logical issues.  Important arguments (e.g., the syndemic of HIV and HPV) are **buried midway** rather than foregrounded.  The logic leading from the **global problem** to the **regional context** and finally to the **study focus** could be clearer.  Several facts are repeated unnecessarily (e.g., HPV causes cervical cancer is stated more than once).  The core **research problem** (the lack of intentional HPV vaccine delivery for girls living with HIV) is only introduced at the end (line 135), but should appear much earlier.  There’s a lack of clear **topic sentences** for each paragraph | Thank you for these comments and clear suggestions. The introduction has been completely restructured, relying largely on the outline you provided in your comments, with a focus on improving the structure and clarity of the logic while retaining its base in the literature. |
| Are there any verbatim quotes you can add to your results section to boost their credibility? It is a qualitative study after all | These have been integrated throughout the results section |
| **This section is overly descriptive, under-analytical.** Much of the text reads like field notes or summaries, rather than synthesized findings. Please adjust | **The results section has been revised to focus more on synthesized findings** |
| Line 208-224; almost no attribution to interview data. missing statements like “Respondents in X country noted...” | This has been addressed throughout the results section to be more clearly attributed to the sources |
| Results could be strengthened by exploring differences by stakeholder type (e.g., government vs NGO vs health worker), or region/district. | This has been included where relevant to the findings throughout the results section |
| Thematic analysis was cited in methods, but the results are not presented as such. | We have chosen to organize the results by study objective, though within each objective, the results have been re-organized to emphasize themes. |
| The discussion is not anchored in any conceptual or theoretical model (e.g., health systems strengthening, implementation science, socio-ecological models).  **Consider** Use a framework like the WHO building blocks or PRISM (used in the methods) to interpret findings and propose recommendations. | This has been updated to more clearly reflect the PRISM framework throughout the discussion section. |
| Line 242-316; Too repetitive. structure is based on countries instead of emergent themes. Long paragraphs with weak transitions and little synthesis. | This section has been comprehensively restructured |
| The first paragraph of the discussion ought to recap study objectives and summarize key findings. Please adjust. | This paragraph has been revised to address this recommendation, thank you. Lines 449-464. |
| Line 451-464; No critical reflection on how to address these issues, especially among GLHIV. Use this section to reflect on how stigma intersects with gender and age. Consider adding a brief reflection on community engagement strategies that have worked elsewhere (e.g., peer educators, youth-led advocacy). | This paragraph was removed from the discussion section to increase relevance and reduce repetition. |
| Line 465-476; Use synthesis: rather than listing what exists, explain how and why these mechanisms are effective. Anchor enablers within a theory (e.g., trust, continuity of care, peer norms). | The discussion section is now more clearly grounded in the PRISM model to better illustrate how these factors contribute to the levels of HPV vaccination in each country |
| Line 477-479; Lacks critical appraisal of feasibility or sustainability. Reflect on whether such strategies are scalable and equitable. Mention contextual adaptation; what works in Eswatini may not work in Malawi. | This has been added, particularly focusing on the importance of adaptation in lines 524-527: “This type of service integration is likely to be feasible across other high-burden HIV and cervical cancer countries and is currently being tested in Zambia, but may require tailoring to country contexts and sufficient resource allocation for scale-up and sustainability” |
| This section can be synthesized better. Also add study strengths | This section has been revised based on comments from both reviewers. Strengths are described in lines 551-556. |
| Some paragraphs are long and could be broken down for clarity. Certain sentences are also dense with multiple ideas.  Some ideas are repeated or could be collapsed (e.g., several mentions of ART clinics and Teen Clubs across different paragraphs).  Use more measured academic language. Replace “Countries should…” with “National programs may consider…” or “Evidence suggests…” | Thank you, these comments informed the restructuring of the policy implications section. |
|  |  |
|  |  |
| No mention of **health system constraints** (financing, HR, competing priorities) that may hinder implementation. | Reference to this has been added in lines 484-487 |
| The section sometimes reads like a general policy proposal. It needs stronger anchoring in study findings. Use phrasing like:   - “As evidenced by stakeholder feedback in Eswatini...” - “Our results showed that in Uganda…” - “Health worker perspectives highlighted…” | References to the evidence base have been added throughout the policy implications section |

| Line 130-132; You say that evidence is limited but don’t mention how. I would demonstrate the evidence limitations to back up that claim. | This claim has been rephrased for clarity in lines 146-7 |
| --- | --- |
| It would be useful to summarize the HIV and cervical cancer stats (ASIR, ASMR, projections by 2045) for all 3 countries in a Table | This has been added (Table 1) |
| What is the relevance of Lines 58-59; 75-76; 92-93; 107-108 | These have been consolidated into the revised text |
| Line 139; What is a cross-sectional qualitative approach? | This section has been updated and this phrase removed |
| Line 140; Who are these stakeholders? Give specifics and justification for picking them | This has been included in more specific paragraphs describing the sampling in the methods section |
| Please clarify how sampling was conducted. | This has been updated in the methods section (lines 156-169) |
| The desk review is described, but it's unclear **how findings were integrated** into the analysis (e.g., were they analyzed thematically too?). | A new paragraph describing the desk review has been included (lines 170-180) |
| Line 145; What is this “other grey literature” | This has been addressed |
| Lines 148-152; this paragraph is better situated as the last part of the methods section | This has been addressed |
| Clearly state **how ChatGPT was used** (e.g., was it prompted with summaries, full transcripts, or coded excerpts?). Clarify whether outputs were used **exploratorily or as part of the primary coding process**. | This has been addressed in lines 203-210 |
| Thematic analysis approach is underspecified; No mention of whether **inductive or deductive coding** was used. No mention of **steps followed** (e.g., familiarization, coding, theme generation). | Additional detail has been added to describe this process in lines 195-210 |
| In the current **methods section, there is no mention of trustworthiness**, which is a crucial component in qualitative research to demonstrate **credibility, dependability, confirmability, and transferability** of findings. | A description of the steps taken to address trustworthiness has been included in lines 199-203 |
| Table 1; You speak of partners and health care workers. I asked for this to be better defined in the methods section | Additional detail has been added on the definition of implementing partner in the methods section |
| Line 186-206;This section feels like background or methods, not results. No integration with interview/FGD data. | This has been addressed in the revised results section |
| Line 225-241; Needs more structure. Combine this with the policy section as “Policy and Awareness.” | This has been restructured and the section heading has been updated |
| Line 405; Novel insights? Really? | This has been removed |
| Line 411; Not the place for this. Also this is what did not come out clearly in the gap analysis in the introduction | This information has been moved to the introduction |
| Consider doing away with the subheadings in the discussion. Just discuss one idea per paragraph | Thank you, the revision reflects this suggestion |
| Line 413; This paragraph is descriptive, not analytical and fails to ask why such gaps persist. Misses opportunity to connect to literature on policy implementation failures in LMICs. Discuss power dynamics, health system fragmentation, and siloed programming (e.g., EPI vs HIV programs). Integrate literature on implementation science or health systems bottlenecks. | We have added references to these challenges |
| Line 438-450; Does not distinguish between general HPV barriers and those unique to GLHIV. No analysis of underlying causes (e.g., funding, capacity, stigma). | This has been added for clarity |
