# Supplementary material for: Policies and strategies for HPV vaccination schedule completion in immunocompromised girls, including girls living with HIV: Qualitative insights from Eswatini, Malawi, and Uganda
Source: PLOS Glob Public Health. 2026 Feb 3;6(2):e0004688. doi: 10.1371/journal.pgph.0004688 (PMC12867219; doi:10.1371/journal.pgph.0004688)
Supplement: Attachment — Submitted filename: HAPPI Response to Reviewer comments 10DEC2025.docx [file pgph.0004688.s008.docx]

Dear Reviewers,

I am happy to share the revised manuscript for the paper titled “Policies and strategies for HPV vaccination schedule completion in immunocompromised girls, including girls living with HIV: Qualitative insights from Eswatini, Malawi, and Uganda,” which we revised following a second round of reviewer comments. These comments, like those in the first round, were constructive and helped us to better describe our study and results; we are grateful for the time taken to support this work. Based on these comments, we have developed a new revised version of the manuscript.

Our detailed responses to the comments follow this letter.

Thank you for considering this revised manuscript, and for the constructive collaboration throughout the process. We remain available to discuss or address any further comments or questions.

Sincerely,
**Sonali M. Patel**
On behalf of all co-authors
[*spatel@clintonhealthaccess.org*](mailto:spatel@clintonhealthaccess.org)
Global Vaccines Delivery Team, Clinton Health Access Initiative

| **Comment** | **Response** |
| --- | --- |
| Not sure about the journal guidelines, but don’t you think subheadings in the abstract will make it more readable? | The authors have noted that in this journal, abstracts are typically presented in a single paragraph without subheadings. The abstract was written to reflect the articles in this journal. |
| You mention that, “A document review of national policies and global publications was also conducted”…What are global publications? Are these covered in the methods? | This sentence has been revised to read “A desk review of relevant policy documents was also conducted.” |
| Are there any stand out country specific findings? If yes, please add them in the abstract. | Brief language referring to country-level results has been added (lines 46-47) |
| The gap analysis needs strengthening…In the last paragraph you say “little is known about HPV vaccine coverage, vaccination strategies, or policies for immunocompromised girls”…it would be useful to demonstrate what is already known (if any) with respect to vaccine coverage, strategies/policies among this population. | Beyond WHO recommendations, we have not found any literature around these topics; however, we are aware that this does not mean that no literature exists, and we would be glad to reference any literature the reviewers are aware of. We have added clarifying language to specify that “no peer-reviewed studies on these topics were identified in literature review” |
| It would be useful to state how these documents were obtained. Search of government websites? | This is explained in lines 144-145 “Documents were identified by research team members with familiarity of the subject matter and were requested in each KII.” |
| Be specific about the kind of data you were extracting from these documents, please | We have updated this to read “All identified documents were read by a research team member, who extracted information relevant to the research questions as described above to a memo.” Lines 143 and 144 (the lines referred to in “as described above”) describe the information we looked for in the documents as “information about vaccination schedules, HPV vaccination, GLHIV, or immunocompromised adolescents” |
| Also clarify the recency of the documents you were looking for. Was there a timeline? | No timeline was used for identification of documents, as we wanted to collect all relevant documents. Table 3 includes the publication year of each document included in the study. |
| One pending issue that needs further clarification, is what ethics committee reviewed and approved this work in Uganda, it is mentioned that it was internally reviewed by the Clinton Health Access Initiative’s internal Scientific and Ethical Review Committee, is this the one that approved the study? | Thank you for this comment, we have updated the language for additional clarity and to specify the Ugandan ethics committee in addition to the UNCST. The text now reads “This study was reviewed and approved by the Clinton Health Access Initiative’s internal Scientific and Ethical Review Committee (SERC). It was also approved by the Eswatini Health and Human Research Review Board (EHHRRB089/2024), the Malawi National Health Sciences Research Committee (NHSRC) (24/09/4522), the Uganda National Council for Science and Technology (UNCST) (HS4994ES), and the Makerere University School of Health Sciences REC (MAKSHSREC-2024-744).” |
| I appreciate the addition of quotes to support the narrative. Could you please pull them out from the main text so the stand out? | This has been adjusted. |
| The first paragraph of your discussion, please add the promising strategies to the list of your key findings. | We have added the following sentence: “These promising strategies mainly rely on identification of eligible GLHIV and delivery of HPV vaccines through two key platforms that serve GLHIV - ART clinics and teen clubs.” |
| This section can be summarized better. Be to the point here.  You have several limitations and only one strength. I would recommend limiting yourself to 3 limitations and adding 2 more strengths | Thank you, we have revised this section to be more concise while also addressing a more balanced approach to considering the strengths and weaknesses of the study. This can be found in lines 516-526. |
| You note that transcripts were not transcribed verbatim, how then did you get quotes that were presented in the results section? From summary notes? | This is correct, quotes were taken from the summary notes, which were largely verbatim though not rigorously transcribed. Summary notes were spot-checked against recordings to verify quality. |
